# Discovering Networks of Perturbed Biological Processes in Hepatocyte Cultures

**DOI:** 10.1371/journal.pone.0015247

**Published:** 2011-01-05

**Authors:** Christopher D. Lasher, Padmavathy Rajagopalan, T. M. Murali

**Affiliations:** 1 Genetics, Bioinformatics, and Computational Biology PhD Program, Virginia Polytechnic Institute and State University, Blacksburg, Virginia, United States of America; 2 Department of Chemical Engineering, Virginia Polytechnic Institute and State University, Blacksburg, Virginia, United States of America; 3 Department of Computer Science, Virginia Polytechnic Institute and State University, Blacksburg, Virginia, United States of America; 4 ICTAS Center for Systems Biology of Engineered Tissues, Virginia Polytechnic Institute and State University, Blacksburg, Virginia, United States of America; Fondazione Telethon, Telethon Institute of Genetics and Medicine, Italy

## Abstract

The liver plays a vital role in glucose homeostasis, the synthesis of bile acids and the detoxification of foreign substances. Liver culture systems are widely used to test adverse effects of drugs and environmental toxicants. The two most prevalent liver culture systems are hepatocyte monolayers (HMs) and collagen sandwiches (CS). Despite their wide use, comprehensive transcriptional programs and interaction networks in these culture systems have not been systematically investigated. We integrated an existing temporal transcriptional dataset for HM and CS cultures of rat hepatocytes with a functional interaction network of rat genes. We aimed to exploit the functional interactions to identify statistically significant linkages between perturbed biological processes. To this end, we developed a novel approach to compute Contextual Biological Process Linkage Networks (CBPLNs). CBPLNs revealed numerous meaningful connections between different biological processes and gene sets, which we were successful in interpreting within the context of liver metabolism. Multiple phenomena captured by CBPLNs at the process level such as regulation, downstream effects, and feedback loops have well described counterparts at the gene and protein level. CBPLNs reveal high-level linkages between pathways and processes, making the identification of important biological trends more tractable than through interactions between individual genes and molecules alone. Our approach may provide a new route to explore, analyze, and understand cellular responses to internal and external cues within the context of the intricate networks of molecular interactions that control cellular behavior.

## Introduction

The liver is one of the important organs in our bodies, playing a vital role in glucose homeostasis, the synthesis of bile acids for the metabolism of cholesterol, and the secretion of proteins to aid clotting [1]. Additionally, the liver is primarily responsible for the detoxification of foreign substances, including a variety of environmental toxicants, alcohol, cigarette smoke, and drugs [1]. Hepatocytes are the principal cells in the liver, comprising over 80% of its mass and performing several characteristic functions of this organ. Liver culture systems such as hepatocyte monolayers (HMs) and collagen sandwiches (CSs) are routinely used to test adverse effects of drugs and environmental toxicants. In HMs, hepatocytes are cultured on a single collagen gel. Such cells progressively lose their phenotypic characteristics over time [2]. In CS systems, hepatocytes are maintained between two collagen gels. Hepatocytes in CS cultures remain stable over extended periods of time, and maintain differentiated hepatic functions [3], [4]. While morphological and physiological characteristics of hepatocytes in CS cultures have been studied extensively, comprehensive transcriptional studies of these culture systems do not appear to have been reported. Therefore, in an earlier study, we performed a systematic temporal study of genome-wide gene expression programs in HMs and in CS cultures over an eight-day period [5]. We used Gene Set Enrichment Analysis (GSEA) [6] to compare the transcriptional programs in the two culture systems. Our results demonstrated that gene expression in hepatocytes in CS cultures steadily and comprehensively diverges from that in HMs [5]. Gene sets up-regulated in CS cultures included several hepatic functions, such as metabolism of lipids, amino acids, carbohydrates, and alcohol, and synthesis of bile acids. Monooxygenases such as Cytochrome-P450 enzymes did not show any change between the culture systems after one day, but exhibited significant up-regulation in CS cultures after three days and later in comparison to HMs.

This analysis did not consider the fact that a cell's response to its environment is governed by an intricate network of molecular interactions. These interactions dynamically change in response to a myriad of cues. Therefore, discovering the set of molecular interactions that are active in a given cellular context is a fundamental question in computational systems biology [7]. In the current work, we reanalyze the CS-HM transcriptional data in the light of an underlying molecular interaction network. We propose a novel approach called “Contextual Biological Process Linkage Network” (CBPLN) that focuses on computing which processes in the cell are perturbed in a particular context and how these processes are linked to each other. Our approach is predicated on the belief that high-level linkages between pathways and processes make identification of important biological trends more tractable and intuitive than through interactions between individual genes and molecules alone. Our method requires three inputs:

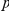
-values representing the statistical significance of the differential expression of each gene (upon comparing a treatment to a control), which we refer to hereafter as *expression* 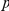 *-values*,
2. a functional or physical interaction network connecting genes and proteins, and
3. a dataset of functional annotations for genes and proteins.

We extend the method developed by Dotan-Cohen *et al.* [8] to detect directed linkages between gene sets in the context of a functional interaction network. Given two biological processes 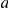 and 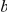 and the sets of genes that are members of each, these authors computed the number of genes annotated by 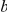 that are themselves not annotated by 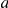 and interact with at least one gene annotated by 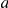. They estimated the statistical significance of this count using the one-sided version of Fisher's exact test. Similar methods developed by Pandey *et al.* [9], [10] for regulatory and physical interaction networks are aimed at discovering chains of significantly linked biological processes.

In this work, we extend the ideas of Dotan-Cohen *et al.* to incorporate gene expression measurements to determine which inter-process links are significantly perturbed between the measured conditions. Informally, we compute a score for a link from process 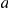 to process 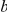 based upon the expression 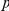-values of pairs of interacting genes, where one gene belongs to process 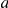 and the other to process 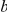. Our score takes estimates of confidence in the interactions into account. High-confidence interactions with highly perturbed incident genes make large contributions to the score. We estimate the statistical significance of the score by computing an empirical distribution of scores under two different hypotheses. The first hypothesis tests the dependence of the score on the particular set of genes annotated by 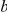, i.e., it asks if we would observe a particular score from process 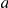 to 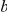 even if we selected the genes annotated by 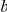 uniformly at random from the set of all annotated genes. This test directly extends the approach used by Dotan-Cohen *et al.* The second hypothesis tests the dependence of the score on the specific interactions in the network, i.e., it asks if we would observe the score from 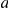 to 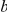 even with an interaction network drawn from a distribution of networks with the same node degrees. Under either hypothesis, we report the significance of the link, after multiple testing correction, as a 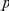-value. Hereafter, we refer to this quantity as the *link* 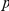 *-value*, to distinguish it from the expression 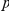-values that are inputs to our method.

## Results and Discussion

### Input Data

#### Gene Expression Data

We used the Affymetrix Rat Genome 230 2.0 GeneChip to measure genomewide transcriptional profiles in rat hepatocytes grown in monolayers and in collagen sandwiches [5]. This dataset is available in MIAME-compliant format in the Gene Expression Omnibus (accession number GSE20659). We marked the day when we deposited the second layer of collagen in CS cultures as day zero. On days one, two, three and eight after deposition of the second layer of collagen, we measured data in triplicate in hepatocytes in each culture system.

#### Functional Linkage Network

Existing databases of protein interactions contain very few experimentally detected Protein-Protein Interactions (PPIs) for rat: seven different widely-used sources [11]–[17] contained a total of just 1,274 non-redundant rat PPIs spanning 974 proteins. Therefore, we decided to use the rat functional linkage network predicted by the STRING system [18]. The interaction type in STRING is a *functional association*, which the authors define as “the specific and meaningful interaction between two proteins that jointly contribute to the same functional process.” Apart from incorporating experimental interaction data, STRING uses multiple methods to predict possible functional linkages including interolog-based interaction transfer, similar transcriptional response across a variety of conditions (co-expression), text-mining, and gene families that share above-random similarities in their evolutionary histories. STRING includes a scheme to score each predicted interaction in the range 150–1000 against a common reference of functional partnership based on the KEGG database [19]. STRING version 8.3, released on May 26, 2010 contains 975,454 predicted interactions among 15,178 rat proteins. We used the subset of these interactions with a weight of at least 500; there were 204,992 such interactions among 9,925 proteins. We selected 500 as a cutoff based on the reasoning that interactions with at least this weight were more likely to connect genes belonging to the same process than to connect genes belonging to different processes. When we further pruned the network to include genes with at least one annotation (see below), we obtained 47,002 interactions among 4,714 genes.

#### Functional Annotations

In our earlier work [5], we used GSEA to compare the two culture systems at each of the four time points; Table 1 lists the contrasts we analyzed. This analysis provided insights into the temporal patterns of up- and down-regulation in the gene sets in the Molecular Signature Database (MSigDB) [6]. In that work, we focused our analysis on gene sets that showed monotonically diverging patterns of expression between CS and HM cultures. In the current paper, we use the curated (c2), motif (c3), and Gene Ontology (c5) collections of gene sets in MSigDB as our set of functional annotations. We focus on establishing linkages among the subset of 18 up-regulated gene sets from the previous study; Table 2 lists these sets along with a short description of each.

**Table 1:** Contrasts analyzed for contextual BPLNs.

Contrast name	Treatment	Control
CS vs. HM 1d	Collagen sandwich 1 day	Hepatocyte monolayer 1 day
CS vs. HM 2d	Collagen sandwich 2 days	Hepatocyte monolayer 2 days
CS vs. HM 3d	Collagen sandwich 3 days	Hepatocyte monolayer 3 days
CS vs. HM 8d	Collagen sandwich 8 days	Hepatocyte monolayer 8 days

**Table 2:** Gene sets from MSigDB selected for our analyses.

MSigDB gene set name	Description
ALCOHOL_METABOLIC_PROCESS	(GO BP) reactions and pathways involving alcohols
CARBOXYLIC_ACID_TRANSMEMBRANE_TRANSPORTER_ACTIVITY	(GO MF) transfer of carboxylic acid across a membrane
CELLULAR_LIPID_METABOLIC_PROCESS	(GO BP) lipid reactions and pathways
GLYCOLYSIS_AND_GLUCONEOGENESIS	participation in glycolysis or gluconeogenesis
HSA00071_FATTY_ACID_METABOLISM	KEGG fatty acid metabolism pathways
HSA00120_BILE_ACID_BIOSYNTHESIS	KEGG bile acid synthesis genes
HSA00220_UREA_CYCLE_AND_METABOLISM_OF_AMINO_GROUPS	KEGG urea cycle and metabolism and amino groups pathways
HSA00251_GLUTAMATE_METABOLISM	KEGG glutamate metabolism pathways
HSA00980_METABOLISM_OF_XENOBIOTICS_BY_CYTOCHROME_P450	KEGG pathways for metabolism of xenobiotics by cytochrome P450
HSA03320_PPAR_SIGNALING_PATHWAY	KEGG PPAR signaling pathway
HSIAO_LIVER_SPECIFIC_GENES	liver tissue genes
HUMAN_TISSUE_LIVER	genes specifically expressed in human liver tissue rather than mouse
MONOOXYGENASE_ACTIVITY	(GO MF) integration of one oxygen atom into a compound
NITROGEN_COMPOUND_CATABOLIC_PROCESS	(GO BP) pathways for breakdown of nitrogenous compounds
NITROGEN_COMPOUND_METABOLIC_PROCESS	(GO BP) pathways for synthesis and breakdown of nitrogenous compounds
NUCLEAR_RECEPTORS	GenMAPP nuclear receptor genes
PEROXISOME	(GO CC) associated with peroxisome
V$HNF1_Q6	genes containing promoter motif for hepatic nuclear factor

### Overview of Results

We considered only those links with a link 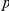-value of at most 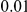, after using the method of Benjamini and Hochberg [20] to adjust for testing multiple hypotheses. We further restricted our attention to pairs of gene sets for which at least 10 genes exclusively in the second set of the pair interacted with genes in the first set, reasoning that fewer interacting genes might not yield robust link 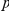-values. We compared the number of links computed by using each hypothesis test. We also compared these values to the number of links in the (context-free) BPLN computed using the method of Dotan-Cohen *et al.* [8]. Tables 3 and 4 display the results of the comparisons.

**Table 3:** Comparison of the properties of the CBPLNs computed by using each hypothesis test.

	Without normalization				With normalization			
	Gene set randomization	Network randomization	Intersection	Jaccard index	Gene set randomization	Network randomization	Intersection	Jaccard index
Day 1	32	21	21	0.66	28	17	17	0.61
Day 2	39	30	30	0.77	33	27	27	0.82
Day 3	75	54	53	0.70	70	52	51	0.72
Day 8	96	81	79	0.81	94	77	75	0.78

There are two groups of columns, one for the results without normalization and another for the results with normalization, where “normalization” refers to results obtained when we deduct the score calculated with average expression values from the observed score. Within each group, the columns titled “Gene set randomization” refer to the number of observed significant links (corrected link 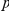-value 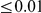) when we construct the null distribution by re-sampling the genes annotated with the gene set 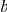; similarly, the columns titled “Network randomization” refer to the number of significant links observed when generating interaction networks with the same node degrees as the original network. The columns titled “Intersection” refer to the number of links significant under both hypothesis tests. The column titled “Jaccard index” contains the ratio of the size of the intersection to the size of the union of the CBPLNs computed by the two hypothesis tests. File S1 contains the statistical significance values for the intersection sizes, as computed by Fisher's exact test.

**Table 4:** Comparison of the number of links in the BPLN to the number of links in the CBPLNs, computed without and with normalization.

	BPLN	Gene set randomization	Intersection	Jaccard index	Network randomization	Intersection	Jaccard index
Without normalization							
Day 1	105	32	32	0.30	21	21	0.20
Day 2	105	39	39	0.37	30	30	0.29
Day 3	105	75	75	0.71	54	53	0.50
Day 8	105	96	93	0.86	81	79	0.74
With normalization							
Day 1	105	28	28	0.27	17	17	0.16
Day 2	105	33	33	0.31	27	27	0.26
Day 3	105	70	70	0.67	52	51	0.48
Day 8	105	94	91	0.84	77	75	0.70

The column titled “BPLN” denotes the number of links in the BPLN. Note that the number of links in the BPLN does not change with the number of days, as the BPLN method does not use gene expression data. The last six columns are divided into two groups of three columns each. The first set of columns compare BPLNs to CBPLNs computed using gene set randomization. The second set of columns compare BPLNs to CBPLNs computed using network randomization. The data in and meaning of columns “Gene set randomization” and “Network randomization” are identical to those in Table 3. The columns titled “Intersection” contains the number of links found to be significant in both the BPLN and the respective CBPLN. The columns “Jaccard index” contains the ratio of the size of the intersection to the size of the union of the BPLN and the respective CBPLN. Statistical significance values for the intersection sizes, as computed by Fisher's exact test, are available in File S1.

Several salient trends emerged. First, in Table 3, irrespective of the hypothesis test used, the number of links increased with time. This phenomenon parallels our earlier observation that the transcriptional programs of hepatocytes in CS cultures steadily diverged from that in HMs. Second, the size of the intersection between the two sets of links also increased with time, as did the Jaccard similarity coefficient of the two sets (i.e., the size of the intersection divided by the size of the union). Further, for each day, the number of links deemed to be significant by both hypothesis tests was itself statistically significant, based on Fisher's Exact Test (see File S1). These trends suggest that once the transcriptional programs of the two culture systems have diverged (day 2 and later), both hypothesis tests find very similar sets of process pairs to be significantly linked at the 0.01 level. However, the number of common links is very close to the number of links identified by the second hypothesis test, indicating that the second test is more conservative than the first in deciding whether a link is statistically significant. We observed similar results when we repeated these analyses with other cutoffs on the link 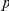-value (0.005, 0.05, and 0.1) (see File S2). Third, normalizing the linkage score (see “Methods”) pruned out a small number of links.

Finally, the overlap between the intersection of the results from both hypothesis tests and the BPLN was small in days 1 and 2 and more substantial in days 3 and 8 (Table 4), although the overlap was still statistically significant by Fisher's Exact Test (see File S1). These data suggest that only a subset of the links in a BPLN may have some relevance to the particular biological conditions being investigated. By incorporating measurements of gene expression, CBPLNs can identify those inter-process links that correspond to the phenotypic differences observed in the two conditions being compared (e.g., hepatocytes in CS versus HM).

**Figure 1:**
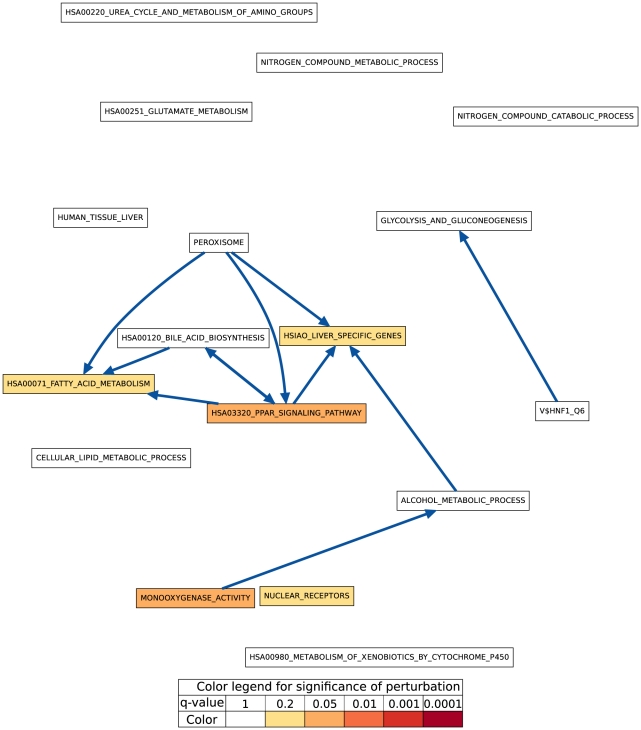
CS vs. HM CBPLN on day 1. In this figure and all other figures displaying CBPLNs, each node is one of the gene sets in Table 2. An edge connects two gene sets whose linkage is determined to be statistically-significant by both hypothesis tests used in computing CBLPNs. The color of a node indicates the statistical significance of its perturbation, as computed by GSEA [6]. The legend mapping colors to ranges of statistical significance appears at the bottom of the figure. We use the same color scheme to indicate the statistical significance computed for a gene set by GSEA and for the significance value computed for a gene by LIMMA. We use this color scheme in all the subsequent figures as well.

**Figure 2 pone-0015247-g002:**
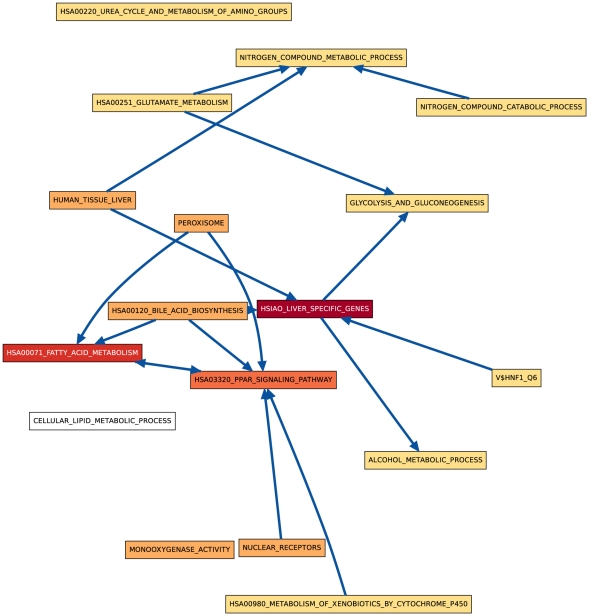
CS vs. HM CBPLN on day 2.

**Figure 3 pone-0015247-g003:**
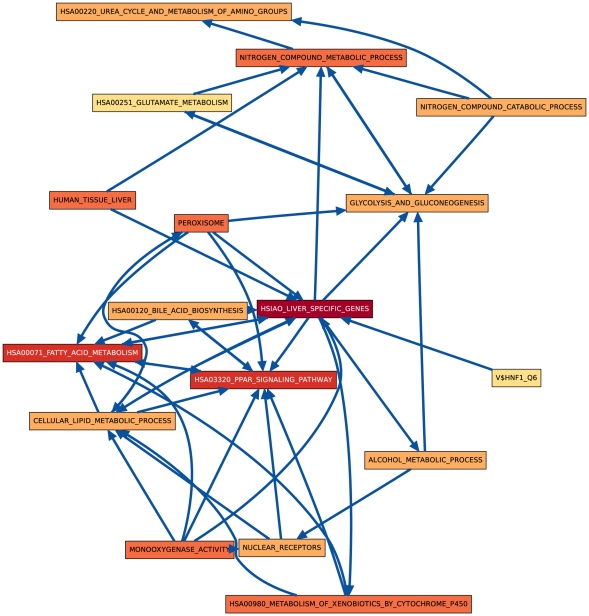
CS vs. HM CBPLN on day 3.

**Figure 4:**
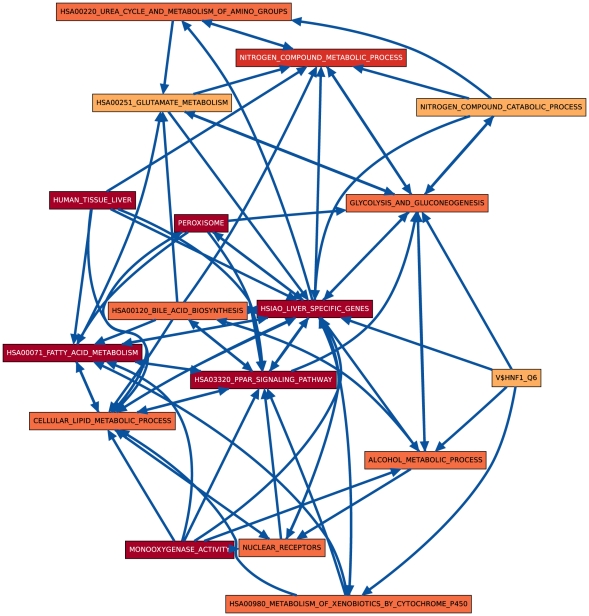
CS vs. HM CBPLN on day 8.

**Figure 5:**
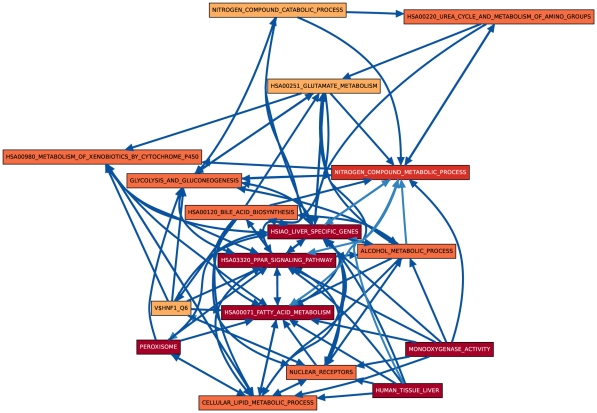
Context-free BPLN, constructed using the approach of Dotan-Cohen *et al.* [**8**]. Colors of gene sets represent perturbation measured by GSEA for CS versus HM at day 8. Note that these perturbation values did not factor into the computation of the BPLN; we display them only for the purpose of visual comparison with Figures 1–4.

Although both hypothesis tests find very similar sets of process pairs to be significantly linked at the 0.01 level, especially in later days, we found that the actual link 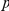-values computed for each process pair were not very highly correlated to each other (see File S3). Based on these results, we decided to consider a linkage between a pair of gene sets only if this link was significant at the 0.01 level with both hypothesis tests with normalization. The resulting CBPLNs are displayed in Figures 1, 2, 3, 4. For reference, we have displayed the BPLN in Figure 5. We discuss the properties of these CBPLNs in the rest of the paper. We focus primarily on the day 8 CBPLN (Figure 4), noting that many of the features we discuss are also apparent in the day 3 CBPLN (Figure 3). When we discuss some pairs of linked gene sets, we refer to the underlying functional interaction network connecting the genes in those sets. We start by discussing properties of liver-specific genes, focusing particularly on the regulation of these genes by the transcription factor HNF1. Then, we discuss the role of lipid homeostasis and bile acid synthesis in the liver. Finally, we summarize the different interpretations of the links in CBPLNs. We stress that the formulation of linkage between processes 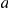 and 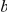 is asymmetric. Hence, by definition, links in the CBPLN are directed, i.e., a CBPLN may contain a link between 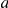 and 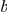 and between 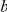 and 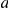.

### Liver Specific Genes

The 251 genes in the HSIAO_LIVER_SPECIFIC_GENES gene set are expressed selectively in the liver, as determined by Hsiao *et al.* [21] from a compendium of gene expression in normal human tissues created with the goal of defining a reference for basic organ systems biology. Genes in this set are members of a spectrum of biological processes, including fatty acid metabolism, metabolism of xenobiotics, blood coagulation, and response to wounding. Not surprisingly, this gene set occupies a central place in the CBPLN on day 8 (Figure 4); it has the highest number of outgoing and incoming links. Outgoing links include connections to glycolysis and gluconeogenesis, alcohol metabolism process, metabolism of xenobiotics by cytochrome P450s, the Peroxisome Proliferator-Activated Receptor (PPAR) signaling pathway, lipid metabolic processes, the urea cycle, and bile acid biosynthesis, among others. In turn, the gene sets such as V$HNF1_Q6 and NUCLEAR_RECEPTORS are linked to HSIAO_LIVER_SPECIFIC_GENES. Some links involving HSIAO_LIVER_SPECIFIC_GENES are unidirectional on day 2 or day 3 (Figures 2 and 3) but bidirectional on day 8 (Figure 4), e.g., to HSA03320_PPAR_SIGNALING_PATHWAY and metabolism of fatty acids, bile acids, and alcohol. Such features suggest that CBPLNs may be representing cellular signals emanating from a subset of liver specific genes to other processes and subsequent feedback from the other processes to liver specific genes. Overall, these results suggest that CBPLNs can assist in the sub-division of liver-specific genes into more refined categories, based not only on the functions of the genes themselves, but also on how they are regulated and what other processes they may control. We discuss one specific link next that illustrates this property.

### Liver Specific Gene Sets Regulated by HNF1

Hepatic nuclear factor 1 (HNF1), also known as albumin proximal factor, is a transcription factor required for the expression of several liver-specific genes including albumin [22]. The protein functions as a homodimer and binds to the inverted palindrome 5′-GTTAATNATTAAC-3′. The promoter regions of genes in the MSigDB set V$HNF1_Q6 match this binding site for HNF1 [23]. In our previous study [5], we noted the monotonic up-regulation of this gene set in CS cultures when compared to HMs. This gene set has an overlap of 25 genes with the gene set HSIAO_LIVER_SPECIFIC_GENES. We concluded that HNF1 monotonically up-regulates the expression of liver-specific genes in CS cultures but not in HMs.

**Figure 6:**
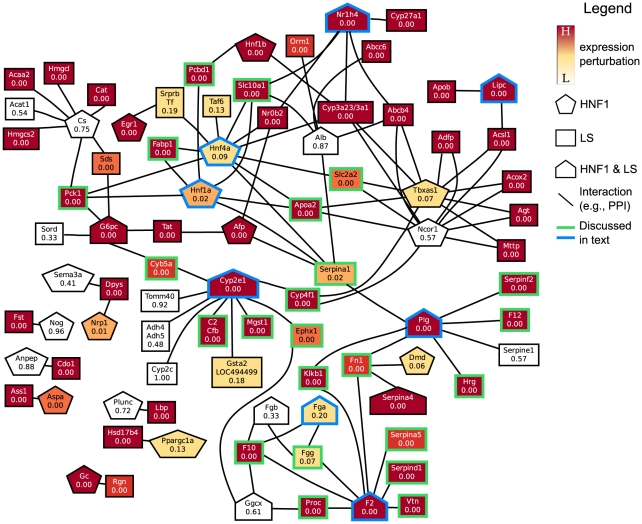
Network of functional interactions resulting in the link between V$HNF1_Q6 and HSIAO_LIVER_SPECIFIC_GENES on day 8. In this and subsequent figures of such networks, each node represents a gene, and its color indicates the statistical significance of its perturbation (up- or down-regulation) in the contrast between CS and HM on the corresponding day. A node's shape represents its membership within the two gene sets: a pentagon represents membership in the first gene set (i.e., V$HNF1_Q6), a rectangle represents membership in the second gene set (i.e., HSIAO_LIVER_SPECIFIC_GENES), and the house shape represents membership in both gene sets. Nodes with blue (respectively, green) borders are those genes in the first (respectively, second) gene set that we mention or discuss in the text. An edge connecting two nodes represents a functional interaction as predicted by STRING. To increase clarity, we do not display interactions between genes within the same set. Abbreviations: HNF1: annotated with V$HNF1_Q6, LS: annotated with HSIAO_LIVER_SPECIFIC_GENES.

CBPLNs assist us in elaborating upon these earlier observations. We studied the link between V$HNF1_Q6 and HSIAO_LIVER_SPECIFIC_GENES in the day 8 CBPLN by examining the functional interactions in the STRING database connecting genes in V$HNF1_Q6 to genes in HSIAO_LIVER_SPECIFIC_GENES. Figure 6 displays a layout of this network. Visual examination of Figure 6 indicates that the linkage between these two gene sets is driven by the genes F2, Plg, CYP2E1, Nr1h4, Lipc, and their interactors, with weaker contributions arising from Hnf1a and Hnf4a. Note that F2, Plg, CYP2E1, Nr1h4, and Lipc are members of both gene sets while Hnf1a and Hnf4a are members of V$HNF1_Q6. We discuss a subset of these proteins next, highlighting liver-specific processes they participate in.

#### HNF1a and HNF4a

Hepatocyte nuclear factor 4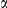 (Hnf4a) is a nuclear receptor implicated in the regulation of numerous genes associated with hepatic function [24]–[26], gluconeogenesis [27], and activation of the metabolism of xenobiotics, including drugs and pharmaceuticals [28]. It is known that both the HNF4 protein and HNF1 protein can transactivate the *HNF1* gene [29]. Although both genes are not very highly up-regulated, their interactions with liver-specific genes Apoa2, Serpina1, Pcbd1, Slc2a2, Slc10a1, Fabp1, and Pck1 suggest the activation of many liver-related pathways.

#### Blood clotting (Plg and F2)

Plasminogen (Plg) is a secreted protein that is proteolysed to plasmin and angiostatin. Plasmin dissolves fibrin in blood clots while angiostatin inhibits angiogenesis. In Figure 6, the significantly up-regulated genes that Plg interacts with include the serpin peptidase inhibitors Serpina1 and Serpinf2, kallikrein B (Klkb1), and coagulation factor XII (F12). Another important protein in Figure 6 is the prothrombin precursor (Coagulation factor II, F2), which interacts with F10, Fga, Fgg, Fn1, Proc, Serpina5, Serpind1, and Vtn. Most of the interactions involving Plg and F2 have been included in STRING via the KEGG pathway for complement and coagulation cascades [19]. The complement system and blood coagulation are a closely interacting pair of proteolytic cascades in blood plasma that are activated after injury [30]. The blood coagulation cascade culminates in the formation of thrombin, the enzyme responsible for the conversion of soluble fibrinogen to the insoluble fibrin clot.

#### Metabolism of xenobiotics (CYP2E1)

Cytochrome P450, family 2, subfamily E, polypeptide 1 (CYP2E1) encodes a member of the cytochrome P450 superfamily of enzymes. Cytochrome P450s proteins are monooxygenases, which carry out the liver's prominent role in xenobiotic metabolism and synthesis of cholesterol, steroids and other lipids. CYP2E1 is an important member of this family, implicated in the metabolism of exogenous compounds such as benzene, carbon tetrachloride, ethylene glycol, and substances found in cigarette smoke as well as endogenous compounds including ethanol, acetone, and acetal [31]–[33]. In Figure 6, CYP2E1 interacts with C2, Cyb5a, CYP4F1, Ephx1, and Mgst1. The interactions of CYP2E1 with Cytochrome P450 4F1 (CYP4F1), Epoxide hydrolase 1 (Ephx1), and Microsomal glutathione S-transferase 1 (Mgst1) are included in the KEGG pathways for metabolism of xenobiotics by Cytochrome P450s and for Arachidonic acid metabolism, which are sources of interactions for STRING. Further support for the role played by CYP2E1 comes from the links to HSA00980_METABOLISM_OF_XENOBIOTICS_BY_CYTOCHROME_P450 from V$HNF1_Q6 and HSIAO_LIVER_SPECIFIC_GENES in the day 8 CBPLN (Figure 4). These links are mediated by the functional interactions between CYP2E1 and members of the alcohol dehydrogenase and glutathione s-transferase gene families (data not shown).

### Lipid Homeostasis and Bile Acid Synthesis

**Figure 7:**
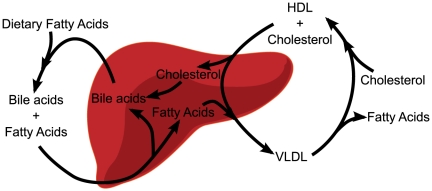
The liver regulates two tightly coupled pathways: bile acid synthesis and fatty acid metabolism. Abbreviations: VLDL: very low-density lipoprotein, HDL: high-density lipoprotein.

**Figure 8:**
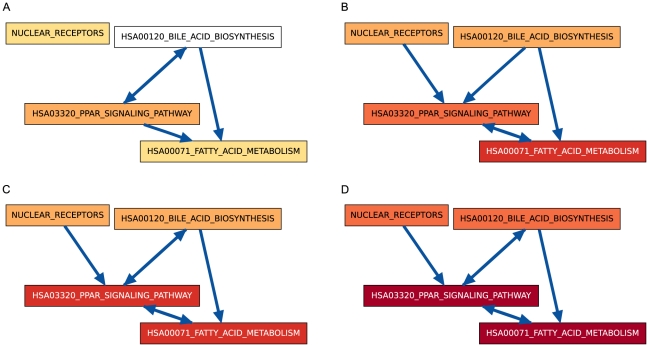
Subgraphs of the CBPLNs involving nuclear receptors and the PPAR signaling, bile acid biosynthesis, and fatty acid metabolism pathways, on days 1 (A), 2 (B), 3 (C), and 8 (D).

Two of the most important functions that hepatocytes in the liver carry out are lipid homeostasis and bile acid synthesis. These two functions are intrinsically linked. As illustrated schematically in Figure 7, the liver produces bile acids, which are secreted into the small intestine, where they allow for breakdown of dietary fats and uptake of fatty acids. Subsequently, the liver re-mobilizes these fatty acids throughout the body via lipoproteins [34]. Lipoproteins circulate fatty acids and cholesterol through the body in a cycle that begins with the liver's secretion of fatty acid-rich very low-density lipoproteins (VLDLs) and ends with the liver's uptake of cholesterol-rich high-density lipoproteins (HDLs) [35]. The liver then recycles these cholesterols or converts them into bile acids. Our results capture the high-level relationships between these processes, as displayed in the sub-CBPLNs involving nuclear receptors, the PPAR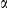 signaling pathway, bile acid biosynthesis, and fatty acid metabolism (Figures 8A–8D).

Before we examine some of these links in more detail, we stress that the links in CBPLNs (e.g., the bi-directional links between HSA03320_PPAR_SIGNALING_PATHWAY and HSA00120_BILE_ACID_BIOSYNTHESIS) must be interpreted with caution. Both HSA03320_PPAR_SIGNALING_PATHWAY and HSA00120_BILE_ACID_BIOSYNTHESIS are up-regulated in CS cultures in contrast to HMs (Fig. 8C and Fig. 8D). Bile acids directly induce the expression of PPAR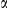 [36], which supports interpreting the observed link from HSA00120_BILE_ACID_BIOSYNTHESIS to HSA03320_PPAR_SIGNALING_PATHWAY as a regulatory one. On the other hand, although it is tempting to infer that the reverse of that link, from HSA03320_PPAR_SIGNALING_PATHWAY to HSA00120_BILE_ACID_BIOSYNTHESIS, also implies the PPAR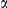 pathway up-regulates bile acid biosynthesis, such a conclusion may be incorrect. Since the up-regulation trends arise from the comparison of CS cultures to HMs, it is possible that bile acid production in CS cultures is constant (or even decreasing) over time and that bile acid levels in HMs are decreasing. In fact, when we compare the expression values of these two gene sets exclusively within the CS cultures, we observe that there is no statistically significant change between the expression levels of the bile acid biosynthesis genes between days 3 and 8, and that there is a barely statistically significant up-regulation of the genes in the PPAR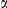 signaling pathway between the same two days (data not shown). Moreover, PPAR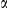 has been shown to directly inhibit production of Cholesterol 7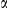-hydroxylase (CYP7A1) [37], [38]. CYP7A1 is the rate-limiting enzyme in the classical pathway of bile acid synthesis from cholesterol [35]. Therefore, while we can conclude from the CBPLN that HSA03320_PPAR_SIGNALING_PATHWAY may regulate HSA00120_BILE_ACID_BIOSYNTHESIS, the mode of regulation (e.g., induction or inhibition) requires more detailed study.

We also note modest changes in the interconnections between the gene sets in Figures 8A–8D over the time-course. One example is the disappearance of the link from HSA03320_PPAR_SIGNALING_PATHWAY to HSA00120_BILE_ACID_BIOSYNTHESIS from day 1 to day 2, followed by the reappearance of this link at day 3. We attribute this behavior to a spurious report of the link as significant at day 1, since we believe our methods may be over-sensitive when very few genes are significantly perturbed in a given contrast (as was the case for day 1). We are currently investigating ways to improve the robustness of our methods in reporting links for such scenarios.

**Figure 9:**
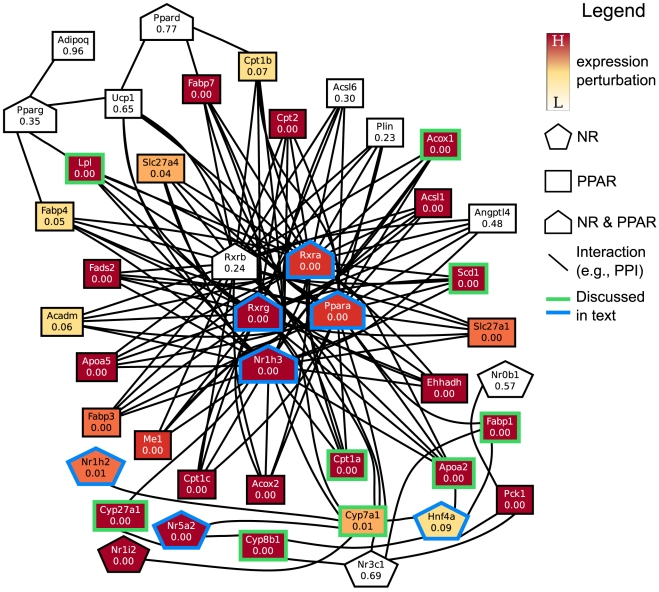
Network of functional interactions resulting in the link between NUCLEAR_RECEPTORS and HSA03320_PPAR_SIGNALING_PATHWAY on day 8. Abbreviations: NR: annotated with NUCLEAR_RECEPTORS, PPAR: annotated with HSA03320_PPAR_SIGNALING_PATHWAY.

**Figure 10:**
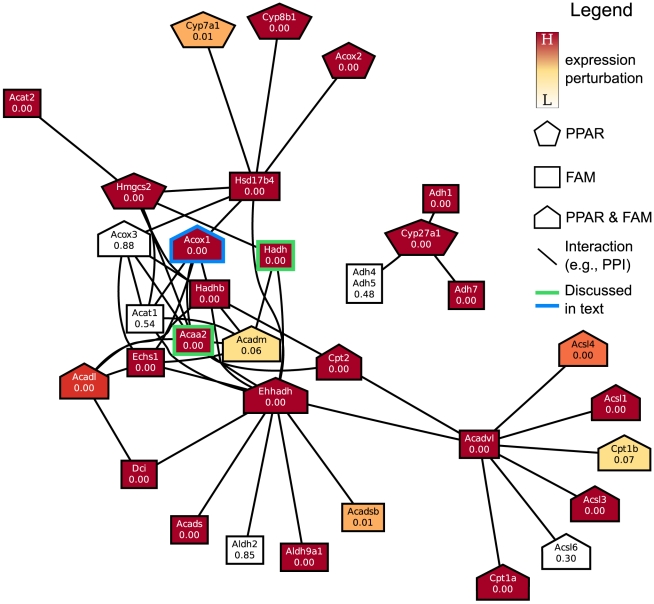
Network of functional interactions resulting in the link between HSA03320_PPAR_SIGNALING_PATHWAY and HSA00071_FATTY_ACID_METABOLISM on day 8. Abbreviations: PPAR: annotated with HSA03320_PPAR_SIGNALING_PATHWAY, FAM: annotated with HSA00071_FATTY_ACID_METABOLISM.

Two other noticeable changes over the time series have immediate biological interpretations. First, the link from NUCLEAR_RECEPTORS to HSA03320_PPAR_SIGNALING_PATHWAY appears at day 2, which we interpret as a regulatory relationship reflected in the underlying functional interaction network and the corresponding up-regulation of the two gene sets. Second, the link from HSA00071_FATTY_ACID_METABOLISM to HSA03320_PPAR_SIGNALING_PATHWAY also appears at day 2, which we interpret in light of feedback in the fatty acid metabolic pathway. In the rest of this section, we discuss the linkages between these three gene sets, anchoring our discussing on the underlying functional interaction networks on day 8 (Figures 9 and 10). We divide our discussion into three parts: interactions of nuclear receptors with cytochrome P450 enzymes, the role played by PPAR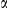, and the regulation of fatty acid metabolism.

#### Interactions of nuclear receptors with cytochrome P450s

In Figure 9, the nuclear receptors that contribute to the linkage between NUCLEAR_RECEPTORS and the HSA03320_PPAR_SIGNALING_PATHWAY are Hepatocyte Nuclear Factor 4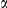 (Hnf4a), Liver Receptor Homolog-1 (Nr5a2/Lrh1), Liver X Receptor 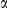 (Nr1h3/Lxra), PPAR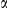, Nuclear Orphan Receptor (Nr1h2/OR-1), and Retinoic acid receptors 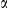, 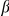, and 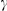 (RXRa and RXRb, RXRg). The dense network of interactions involving PPAR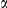, RXRa, RXRb, and Nr1h3 have been incorporated into STRING from curated pathway databases such as REACTOME [11].

All these nuclear receptors exhibit increasing perturbation over time, and interact with CYP7A1, a cytochrome P450 enzyme that is a member of the PPAR signaling pathway. Note that CYP7A1 itself shows no significant perturbation until day 8. We discuss the support in the literature for a subset of the interactions with CYP7A1. HNF4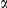 has been shown to bind to the promoter regions of CYP7A1, resulting in up to a nine-fold increase in production of the CYP7A1 protein *in vitro* [39]. The literature suggests tenuous regulatory connections between liver receptor homolog 1 (LRH-1, or Nr5a2) and CYP7A1. *In vitro* studies have shown that Nr5a2 both promotes and represses the expression of CYP7A1 [40], [41]. In a recent study, a knockout of *Lrh-1* (*Nr5a2*) performed selectively in cells that developed into mouse hepatocytes demonstrated that the absence of Nr5a2 had little effect on expression of *CYP7A1* [42].

Liver X receptors regulate cholesterol and lipid homeostasis in multiple tissues via two isoforms: LXR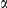 (Nr1h3), which is highly expressed in liver, and LXR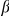 which is more abundant in adipose tissue, gut, kidney, and macrophages [43]. In contrast to the connection between LRH-1 and CYP7A1, LXR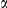 is well known to activate transcription of CYP7A1 in the presence of cholesterol [44]. Thus, it is surprising that we did not observe significant perturbation in expression of *CYP7A1* until day 8. However, *in vitro* studies indicate that CYP7A1 protein exhibits low turnover [35], raising the possibility that the hepatocytes in both cultures had ample amounts of the proteins up to day 3.

Another set of contributions to the linkage between these two gene sets come from interaction of the nuclear receptors Hnf4a and Nr5a2/Lrh1 with sterol 12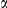-hydroxylase (CYP8B1), a member of the PPAR signaling pathway. CYP8B1 catalyzes a fate-determining reaction in which cholesterol is ultimately converted into the primary bile acid cholic acid, rather than chenodeoxycholic acid [35]. The study of selective knockout of *Lrh-1* (*Nr5a2*) in mice [42] showed that, in contrast to the effect on the expression of *CYP7A1*, the knockout caused a significant drop in expression of *CYP8B1*, demonstrating a very strong regulatory relationship between Nr5a2 and *CYP8B1* [42]. Additionally, strong experimental support for Hnf4a promotion of *CYB8B1* expression exists [45]. Thus, the expression of *CYP8B1* also increases over time, although it lags the expression of its regulatory receptors Hnf4a and Nr5a2.

Nr5a2 is also predicted to interact with 27-hydroxylase (CYP27A1), a mitochondrial cytochrome P450 enzyme that is responsible for a step in the conversion of cholesterol to approximately 25% of the bile acids in mouse [35]. We observe an increase in the perturbed expression of *CYP27A1* concomitant to but lagging that of *Lrh-1* (*Nr5a2*). The knockout of *Lrh-1* led to significantly decreased expression of *CYP27A1* [42], supporting the interaction of these two genes.

#### The role of PPAR
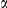


Next, we focus on the role played by PPAR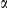 in the linkage between nuclear receptors and the PPAR signaling pathway. PPARs are a class of nuclear receptors responsive to fatty acid ligands. PPARs have been divided among three known subtypes, 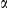, 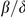, and 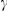, with each subtype occurring in distinct tissues and effecting differing biological responses. Liver cells express PPAR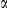, which is responsible for the regulation of fatty acid uptake and catabolism [46], [47]. In our data, only PPAR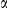 shows increasing expression in CS cultures, compared to HMs; the other PPARs are not significantly different between the two culture systems.

In Figure 9, the significantly perturbed members of the PPAR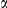 pathway that PPARa interacts with include Scd1, Fabp1, Apoa2, Lpl, Acox1, Cpt1a, and CYP7A1. PPAR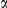 has been shown to promote expression of these genes by binding to their upstream Peroxisome Proliferator Regulatory Element (PPRE) regions as a heterodimer with RXR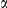 (reviewed in [48]). We note that RXR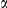 shows significant up-regulation in CS versus HM, as well (Fig. 9). RXR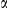 has been shown to be particularly highly expressed in the liver [49]. RXR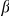, however, tends to have low expression levels across all tissues [49]. The significant up-regulation of RXR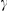 in CS versus HM is somewhat puzzling, given that RXR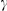 tends to be exclusively expressed in the brain, anterior pituitary, and skeletal muscle [49]–[51], where it is responsible for triglyceride uptake and metabolism [52]. We discuss a subset of the interactions involving PPAR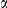 next.

Stearoyl-Coenzyme A desaturase 1 (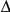
9-desaturase, Scd1) is the main hepatic isoform of SCD. Scd1 helps catalyze the rate-limiting step in the synthesis of monounsaturated fatty acids, particularly the production of palmitoleic acid and oleic acid from palmitic acid and stearic acid, respectively [48], [53]. LXR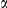 indirectly regulates transcription of Scd1 through activation of transcription of sterol regulatory element binding protein (SREBP) 1c [54], [55], an activator of Scd1 transcription [56], [57]. Additionally, LXR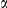 directly activates *Scd1* transcription through an upstream response element [58]. PPAR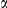 has also been demonstrated to directly activate transcription of *Scd1* [59]. Thus, our observation of increasingly significant changes in expression for LXR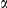 and PPAR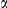, and a similar trend in Scd1, runs in accordance with previous studies.

The interaction of Fatty Acid Binding Protein 1 (Fabp1, L-FABP) with PPAR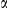 through protein-protein contacts is thought to promote the expression of proteins involved in fatty-acid oxidation and gluconeogenesis [60], [61]. Included among these genes is *Fabp1*. Thus, it regulates its own expression through PPAR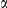.

#### Regulation of fatty acid metabolism by nuclear receptors

The genes in NUCLEAR_RECEPTORS are responsible for initiating cellular responses to a wide variety of conditions and for starting appropriate signal cascades. The nuclear receptors in HSA03320_PPAR_SIGNALING_PATHWAY are the specific subset responsible for initiating the signaling cascade leading to the breakdown of long chain fatty acids [48]. The gene set HSA00071_FATTY_ACID_METABOLISM contains the full contingent of genes responsible for the catabolism of fatty acids. HSA03320_PPAR_SIGNALING_PATHWAY acts as a bridge between the two general classes of genes, NUCLEAR_RECEPTORS and HSA00071_FATTY_ACID_METABOLISM. Figure 9 shows the interactions of individual genes in NUCLEAR_RECEPTORS with those in HSA03320_PPAR_SIGNALING_PATHWAY responsible for the upstream processes of fatty-acid catabolism, including uptake, such as L-FABP (Fabp1) and early-stage fatty-acid 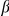-oxidation in the peroxisome, such as acyl-Coenzyme A oxidase 1 (Acox1) [48]. Figure 10 shows the individual genes in HSA03320_PPAR_SIGNALING_PATHWAY that interact with those in HSA00071_FATTY_ACID_METABOLISM responsible for later stages of 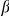-oxidation in the mitochondria, such as acetyl-Coenzyme A acyltransferase 2 (Acaa2) and hydroxyacyl-Coenzyme A dehydrogenase/3-ketoacyl-Coenzyme A thiolase/enoyl-Coenzyme A hydratase 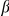 (Hadhb) [48]. Thus, the signals from NUCLEAR_RECEPTORS are transferred to HSA00071_FATTY_ACID_METABOLISM via the subset of nuclear receptors that are members of the PPAR signaling pathway, a chain of events that we are able to recover in the CBPLNs.

### Interpretation of Links in CBPLNs

Keeping the examples of the previous sections in mind, we now discuss how links in CBPLNs might be interpreted.

#### Regulatory relationship

Gene set 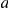 may contain genes whose products regulate genes and/or their products in gene set 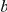. An example is the linkage from NUCLEAR_RECEPTORS to other gene sets such as HSA03320_PPAR_SIGNALING_PATHWAY and genes involved in cellular lipid metabolism; many liver-specific nuclear receptors such as LXR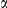 and HNF4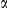 regulate critical hepatic processes.

#### Multi-input motif

Multiple gene sets may link to a gene set 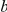, suggesting that the expression of genes in 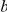 is regulated by genes in multiple other sets. Such a phenomenon is called a “multi-input motif” in the case of a gene being regulated by multiple transcription factors [62]. An example is HSIAO_LIVER_SPECIFIC_GENES and links to this gene set from V$HNF1_Q6 and NUCLEAR_RECEPTORS.

#### Feedback

Links that exist in both directions between 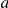 and 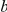 may suggest that 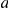 regulates 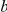 and that 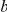 receives a feedback signal from 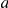. This phenomenon may be observed within CBPLNs when the link is unidirectional at some time points and bidirectional in later time points. A specific example is the linkage between bile acid biosynthesis and HSA03320_PPAR_SIGNALING_PATHWAY, which is unidirectional on day 2 (Figure 8B) but bidirectional on days 3 and 8 (Figures 8C and 8D).

#### Downstream in the signal flow

A link from process 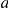 to process 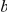 and another from 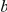 and process 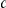 may suggest that 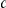 lies downstream of 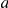. An instance of this feature is the link from NUCLEAR_RECEPTORS to HSA03320_PPAR_SIGNALING_PATHWAY and the link from HSA03320_PPAR_SIGNALING_PATHWAY to HSA00071_FATTY_ACID_METABOLISM in Figure 8B.

#### Multi-functional gene set

A gene set 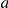 that has many incoming links and/or many outgoing links might be an example of a multi-functional gene set. A prominent example in our CBPLNs is the central HSIAO_LIVER_SPECIFIC_GENES gene set. As we remarked earlier, the links incident on this gene set suggest what other processes the genes in HSIAO_LIVER_SPECIFIC_GENES may regulate or be connected to. Clearly, such a feature depends on how a gene set is defined. For example, many biological processes in the Gene Ontology such as “response to stress” are themselves composed of well-defined and functionally-coherent processes. Similarly, the genes that are perturbed by a particular stimulus may participate in a wide variety of processes. CBPLNs can situate such genes in a rich context within the underlying network of molecular interactions.

### Conclusions

We have presented an approach that represents cellular responses at the granularity of biological processes and connections among them. Our approach extends the work of Dotan-Cohen *et al.* [8] by integrating transcriptional data (the “context”) with functional interaction networks. We focused our analysis on nearly 20 MSigDB gene sets we had identified as up-regulated in hepatocyte cultures in an earlier study. CBPLNs revealed numerous meaningful connections between different biological processes and gene sets, which we were successful in interpreting within the context of liver metabolism. Links and local network features in CBPLNs are generalizations of diverse physiological phenomena such as regulation, feedback, and downstream signal flow from the gene/protein level to the scale of biological processes.

Our approach is a complement to a suite of methodologies that integrate physical, signaling, regulatory, and functional networks with measurements of molecular profiles such as transcriptional, proteomic, or metabolic data to compute the *response network*, which may be defined as the sub-network of interactions that are perturbed in a particular condition. A wide variety of methods have been developed for computing such response networks [63]–[67]. Response networks are typically interpreted by computing which biological processes are enriched in them. In contrast, rather than compute the entire response network, we focus on discovering connections between perturbed biological processes. Since response networks can include genes without any annotations, they can be used to predict biological processes to which unannotated genes belong [68]. In contrast, only genes annotated to some biological process can contribute to CBPLNs. A detailed comparison of CBPLNs to response networks and the development of methods that combine both approaches will be the focus of future research.

Generalizing our approach to the entire spectrum of MSigDB gene sets or to the set of all biological processes in the Gene Ontology raises several interesting challenges. First, gene sets can have considerable overlap, leading to redundant links. Second, scaling this approach up to thousands of gene sets may result in tens to hundreds of thousands of links that are deemed to be statistically significant. This deluge of links will be hard to interpret. Third, it will be challenging to computationally scale our permutation-based sampling to the large number of process pairs we will have to test. We are currently investigating these issues.

In this work, we computed CBPLNs for two conventional hepatocyte culture systems. Three dimensional liver mimics [69], [70] and microscale co-culture systems [71] have shown improved retention of hepatic phenotype over conventional systems. In the future, we plan to apply CBPLNs to liver mimics and co-culture systems in order to obtain insights into the inter-cellular signaling mechanisms that confer improved hepatic phenotype. More generally, our approach may provide a novel route to explore, analyze, and interpret cellular responses to internal and external cues.

## Materials and Methods

### Measuring perturbation from gene expression data

We applied Linear Models for Microarray Data (LIMMA) [72] to the DNA microarray data to compute expression 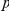-values indicating the differential expression of each gene for each of the four contrasts shown in Table 1.

### Scoring a link between a pair of processes

We first present the approach developed by Dotan-Cohen *et al.* to identify linkages between biological processes [8]. Given an intracellular interaction network for an organism and Gene Ontology annotations for the genes in those networks, Dotan-Cohen *et al.* compute what they term a *Biological Process Linkage Network* (*BPLN*). Informally, given two biological processes, they defined the first process as being *linked to* the second process if genes annotated by the first process interact with a significant number of genes annotated by the second process. By definition, such links are directed. The resulting output of the algorithm by Dotan-Cohen *et al.* is, for each ordered pair of processes, the probability that the first process is linked to the second.

Formally, let 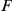 be the set of all biological processes. We seek to ask “Given two processes 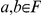, is process 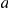 *linked to* process 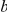
?” More specifically, of the genes that are neighbors of those annotated by 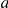, are many more annotated with 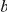 than would be expected by chance? Let 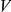 be the set of all genes in an organism. Let 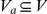 be the set of genes annotated by process 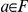, and let the *universe* 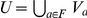, be the set of all genes annotated by at least one process in 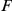. Let 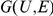 denote an undirected interaction graph where 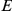 is the set of undirected edges 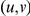, each representing an interaction between genes 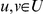. We define the set 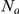 as the set of genes 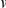 that meet the following criteria:

1. gene 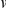 neighbors at least one gene 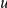 annotated with 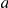

2. gene 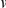 is not annotated with 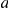.

In other words,
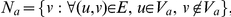
Next, we define 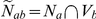, i.e., the set of genes that are neighbors of genes annotated with 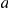, are not annotated with 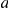 themselves, and are annotated with process 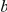. We define the link 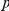-value 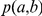 as the probability that, if we selected a set 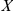 of 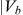 genes uniformly at random from 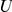, the set 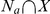 would contain 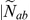 or more genes. We can compute this link 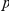-value as the tail of a hypergeometric distribution:
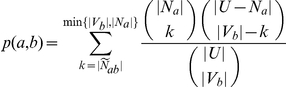
If this link 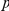-value is significant at some cutoff 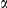, we conclude that process 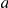 is linked to process 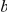.

### Extending the score to include transcriptional data and interaction weights

With this background, we extend the formulation of BPLN to take transcriptional measurements and interaction weights into account. For each interaction 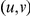 in the graph 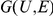, we use 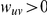 to denote its weight. The larger the weight of an interaction, the larger is our belief that 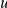 and 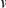 indeed interact functionally in the cell. We define a scoring function 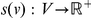 that maps genes to a non-negative real number representing their degree of perturbation in a given biological context (e.g., CS day 8 versus HM day 8). In this work, we compute 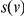 as absolute value of the logarithm of the LIMMA 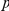-value of the gene. Given processes 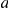 and 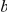, we first define a score 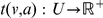.

The function 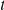 measures the contribution of the neighbors of 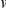 annotated with term 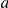 based on their perturbation. Ideally, if at least one neighbor of 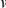 that is annotated with 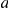 is highly perturbed, we desire that 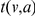 take a high value. On the other hand, if no such neighbor of 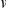 is highly perturbed, we desire that 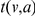 take a small value. Naturally, the weights of the interactions should also play a role in 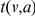. Accordingly, we define
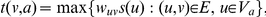
i.e., 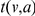 is the maximum weighted score of all neighbors of node 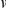 that are annotated with process 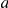.

**Figure 11:**
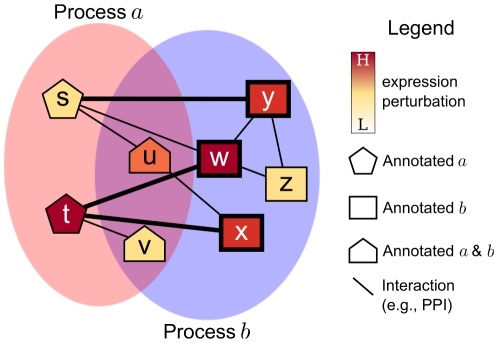
Calculating the links score 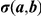 in an example network. Nodes with bold borders (w, x, and y) represent genes in 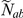. Bold edges indicate the interactions contributing to 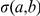.

We define the *contextual linkage score* 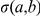 between processes 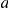 and 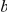 as the following:
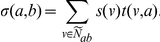

Figure 11 contains a toy example that illustrates these concepts. Thus, a node 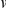 annotated by 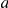 makes a large contribution to the contextual linkage score 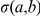 if 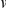 shows a high amount of perturbation in a particular context and if the neighbors of 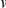 annotated by 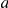 also show a high amount of perturbation. If we have many such nodes 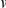, then 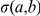 itself will be large. Note that if we set 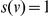 for all 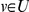 and if all edges have weight 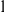, then 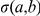 is equal to the size of 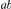, identical to the score computed by the original BPLN algorithm.

In this formulation, some pairs of processes may have a high contextual linkage score even if all genes were perturbed by the same amount. To account for this possibility, we compute a normalized score 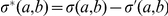, where 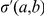 is a background score computed in the same manner as 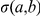, but, after setting the gene perturbation score 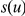 equal to the average expression 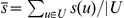 for all genes 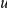 in 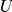. Thus, 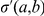 represents the score for the link between processes 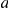 and 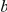 if all genes had the average expression score.

### Assessing the statistical significance of links

Since the contextual linkage score is a weighted generalization of the statistic measured by Dotan-Cohen *et al.* it is unclear how to compute its statistical significance analytically. Therefore, we use two different approaches in order to assess the significance of the observed score 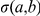 empirically.

1. The first approach is an empirical version of the test performed by Dotan-Cohen *et al.* [8]: what is the probability that we would observe a score 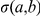 or more if we were to randomly select the nodes annotated with 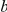
? Specifically, we repeatedly select a set 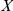 of size 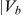 uniformly at random without replacement from 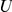 and calculate 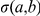 for each of these random selections. After performing the step 10,000 times, we return the fraction of random scores that are larger than the observed value of 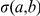 as the link 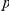-value 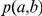.
  Two different processes may annotate some genes in common. To preserve this property even in the random selections of the set 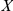 over different processes, we adopt the following approach: we construct a bipartite graph 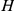 in which a node is a gene or a biological process and an edge connects a gene to a biological process it is annotated with. We randomly permute the labels of the genes in this graph. To generate a random set 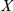 of size 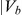, we simply select the genes annotated with 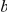 in the bipartite graph with randomized gene labels. These steps create a randomized set of annotations that satisfy two properties: (a) every process annotates the same number of genes as in the original set of annotations, and (b) if 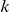 genes are annotated by each process in a set of processes 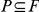, then these processes co-annotate exactly 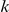 genes in the randomized dataset as well.
2. The second approach accounts for the role played by the interactions between the genes in 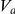 and genes in 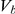. Therefore, we generate a graph 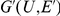 with the property that each node 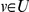 has the same degree in 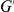 and 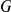. We measure the contextual linkage score between 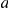 and 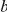 with respect to 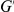. We generate 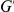 10,000 to build a null distribution for the contextual linkage score, and compute the link 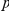-value 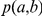 as before.
  To construct 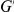, we follow the “edge-swap” approach [73]. We begin with the set of edges 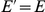 and modify the edges in 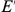 with pairwise edge swaps. For each edge swap, we first select a pair of edges 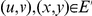. We then select, with equal probability, either 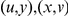 or 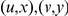 (i.e., the edges created by swapping the endpoints of the original pair of edges) as a candidate edge pair. If either candidate edge already exists in 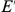 or creates a self-loop, we retain the original pair of edges in 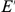, i.e., we do not perform the edge swap. Otherwise, we remove the original edges 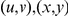 from 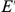 and insert the new edges into 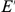. In total, we perform 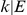 edge-swap events to create a randomized graph 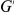, where 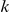 is a user-defined parameter. In this work we used 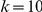.

We use the method of Benjamini and Hochberg [20] to correct for testing multiple hypotheses, while ensuring that the corrected link 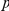-values are monotonic [74]. For either approach, if 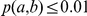, we say that term 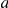 is linked to 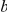 in the given biological context.

## Supporting Information

File S1 File S1 is in tab-separated values format. It contains results of comparisons on the number of links identified to be significant under the two hypothesis tests, as well as under the original BPLN algorithm by Dotan-Cohen, *et al.* [8], which does not consider gene expression data. Six tables are given for different pairwise comparisons of hypothesis tests. In the table headers, “gene set” indicates testing the significance of a link when compared to a distribution of scores calculated from randomized annotations, “network” indicates testing the significance of a link when compared to a distribution of scores calculated from a randomized network, “normalization” indicates the scores were normalized by deducting the score calculated for averaged expression, and “bpln” indicates testing the significance using the original BPLN algorithm. Column headers of tables are defined as follows: “day” indicates the time point of the contrast; “in both” indicates the number of links found to be significant in the two compared hypothesis tests (e.g., gene set randomization and network randomization); “first only” indicates the number of links found significant under the first hypothesis test (e.g., gene set randomization); “second only” indicates the number of links found significant under the second hypothesis test (e.g., network randomization); “neither” indicates the number of links not found significant under either hypothesis test; “intersection significance” indicates the significance of the number of links found significant under both hypothesis tests versus what would be expected by chance, as assessed under Fisher's Exact Test.
(TSV)

File S2 File S2 is in tab-separated values format. It contains results of comparisons between the two different hypothesis tests, as well as the original BPLN algorithm by Dotan-Cohen, *et al.* [8]. Four sets of tables appear indicating the comparison of results at different cutoffs for considering a link to be significant. The header of each set indicates the cutoff used: 0.005, 0.01, 0.05, or 0.1. In each set of tables, the first set is the pairwise comparison under the two hypothesis testing methods of gene set randomization and network randomization, using normalization. The column headers for this table are defined as follows: “Gene set randomization normalized” indicates the number of links found to be significant under gene set randomization with normalization; “Network randomization normalized” indicates the number of links found to be significant under network randomization with normalization; “Intersection” indicates the number of links found significant under both forms of randomization; and “Jaccard index” indicates the ratio of the size of the intersection of the sets of links significant under the two tests to the size of their union. In the second table of each set, the results under the original BPLN algorithm are compared to those of the two hypothesis tests. The column headers for this table are defined as follows: “BPLN” indicates the number of links found significant under the original BLPN algorithm; “Gene set randomization normalized” and “Network randomization normalized” are identical to the first table; “Intersection” indicates the number of links found significant under the original BPLN algorithm and the respective hypothesis test (e.g., under gene set randomization); “Jaccard index” indicates the ratio of the size of the intersection of the sets of links found significant under BPLN and the respective hypothesis test to the size of the union.
(TSV)

File S3File S3 contains scatter plots of link 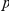-values for links found to be significant (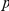-value 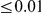) by least one of the hypothesis tests (based on gene set randomization or on network randomization) with normalization. Each plot corresponds to a single day. Each point on a plot corresponds to one pair of processes, with the 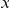-coordinate being the 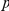-value from gene set randomization and 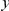-coordinate representing the 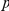-value from network randomization. In each plot, both axes are on a logarithmic scale.(TIFF)
