# Supplementary figures and images for: Discovering Networks of Perturbed Biological Processes in Hepatocyte Cultures
Source: PLoS One. 2011 Jan 5;6(1):e15247. doi: 10.1371/journal.pone.0015247 (PMC3016309; doi:10.1371/journal.pone.0015247)

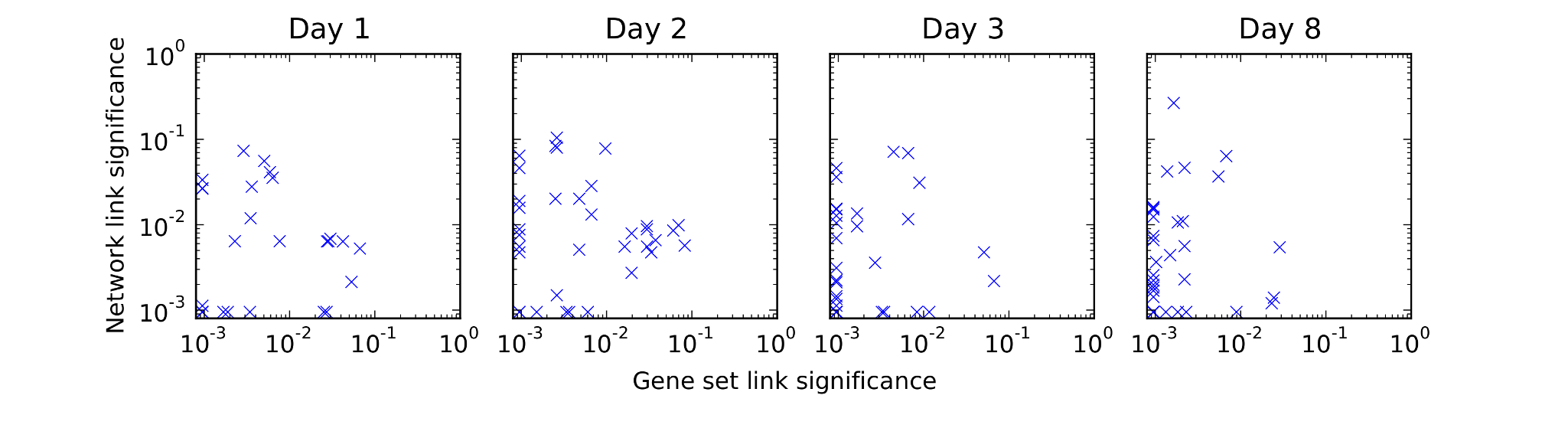

Supplement: File S3 — contains scatter plots of link -values for links found to be significant (-value ) by least one of the hypothesis tests (based on gene set randomization or on network randomization) with normalization. Each plot corresponds to a single day. Each point on a plot corresponds to one pair of processes, with the -coordinate being the -value from gene set randomization and -coordinate representing the -value from network randomization. In each plot, both axes are on a logarithmic scale. (TIFF) [file pone.0015247.s003.tiff]
